# The effect of geometry on three-dimensional tissue growth

**DOI:** 10.1098/rsif.2008.0064

**Published:** 2008-03-18

**Authors:** Monika Rumpler, Alexander Woesz, John W.C Dunlop, Joost T van Dongen, Peter Fratzl

**Affiliations:** 1 Department of Biomaterials, Max Planck Institute of Colloids and Interfaces14424 Potsdam, Germany; 2 Energy Metabolism Research Group, Department of Organelle Biology, Biotechnology and Molecular Ecophysiology, Max Planck Institute for Molecular Plant Physiology14424 Potsdam, Germany

**Keywords:** three-dimensional tissue formation, scaffold channels, bone tissue engineering, curvature-driven growth, osteoblasts

## Abstract

Tissue formation is determined by uncountable biochemical signals between cells; in addition, physical parameters have been shown to exhibit significant effects on the level of the single cell. Beyond the cell, however, there is still no quantitative understanding of how geometry affects tissue growth, which is of much significance for bone healing and tissue engineering. In this paper, it is shown that the local growth rate of tissue formed by osteoblasts is strongly influenced by the geometrical features of channels in an artificial three-dimensional matrix. Curvature-driven effects and mechanical forces within the tissue may explain the growth patterns as demonstrated by numerical simulation and confocal laser scanning microscopy. This implies that cells within the tissue surface are able to sense and react to radii of curvature much larger than the size of the cells themselves. This has important implications towards the understanding of bone remodelling and defect healing as well as towards scaffold design in bone tissue engineering.

## 1. Introduction

Cells react to the chemistry, geometry (Chen *et al*. 1997) and mechanics (Discher *et al*. 2005) of their local environments. In addition to the well-studied response of cells to biochemistry, the influence of mechanical parameters is well described for mesenchymal stem cells where it is possible to control the differentiation of these cells through substrate stiffness. Neuronal cells, for example, are formed on soft substrates, muscle cells on stiffer substrates and osteonal cells on more rigid matrices (Engler *et al*. 2006). The focal adhesion sites of the cells become more stable and the cell cytoskeleton more organized with increasing substrate stiffness, enabling larger forces to be transmitted (Discher *et al*. 2005). The cells appear to probe their local environment through the transmission of forces from the environment via the focal adhesion sites attached to the cytoskeleton (Balaban *et al*. 2001) and seem to be able to sense and transmit forces over long distances relative to their size (Bischofs & Schwarz 2003). The geometric arrangement of the local environment is also a critical factor in determining cell behaviour. It has been shown, for example, that both the chemical composition and the sub-cellular organization of focal adhesion molecules differ for cells grown in a three-dimensional matrix compared with those grown on a flat plate in two dimensions (Cukierman *et al*. 2001; Cavalcanti-Adam *et al*. 2007). On the micrometre scale, the geometry of adhesive regions controls the cell shape and the spreading of cells across a substrate (Chen *et al*. 1997). The cells also respond to variations in geometry on the nanoscale, and substrates patterned with different densities of integrin-coated nanodots show differences in the dynamics of focal adhesion and cell motility (Cavalcanti-Adam *et al*. 2007).

The influence of surface geometry is well studied for the behaviour of single cells, but no data are available for the collective behaviour of groups of cells, namely at the tissue level. Such data are essential in tissue engineering to optimize the support for new tissue formation in artificial replacement materials. Scaffolds for tissue engineering act as a physical support structure to replace tissue removed after injury or cancer ablation, and ideally should regulate biological events such as cell proliferation, viability and intracellular signalling (Geiger *et al*. 2001; Geiger & Bershadsky 2002). Bone replacement materials, for example, should have an open porosity with channels of the order of a few hundred micrometres diameter. This not only allows for the migration of single cells into the scaffold but also provides sufficient space for the formation and growth of new tissue with a cellular and structural organization similar to that seen *in vivo* (Woesz *et al*. 2005; Rumpler *et al*. 2007). Studies investigating the effect of pore size at the micrometre level have focused on cell attachment, migration and cell division with respect to biocompatibility (Van Eeden & Ripamonti 1994). Pore channels in scaffold materials can vary between round and irregular cross-sections (Jin *et al*. 2000; Habibovic *et al*. 2005), but the effect of pore shape on tissue formation has not yet been investigated systematically. An understanding of the effect of geometry on tissue growth could assist in the optimization of porous scaffolds for tissue engineering as well as help improve understanding the processes of bone remodelling and healing.

This paper investigates a model system for tissue growth consisting of three-dimensional hydroxylapatite (HA) plates of controlled architecture placed within a culture of murine osteoblast-like cells. These cells are known to undergo differentiation from an immature pre-osteoblastic to a mature osteoblastic phenotype *in vitro*, accompanied by the expression of characteristic marker proteins at each stage of development (Quarles *et al*. 1992). Furthermore, they are capable of building an extracellular matrix consisting of densely packed and well-organized collagen fibrils with representative non-collagenous matrix proteins known from bone tissue (Choi *et al*. 1996; Rumpler *et al*. 2007). The aim of this paper is to investigate the impact of geometrical features (channel shape and size) on new tissue formation *in vitro*.

## 2. Material and methods

### 2.1 Production of the HA plates

HA plates (2 mm thick) containing four channel shapes (triangular, square, hexagonal and circular) and three channel sizes (perimeter *P*=3.14, 4.71 and 6.28 mm) were produced by slurry casting. Casting moulds were designed using computer-aided design (CAD) software (Pro/Engineer PTC, USA) and produced using a three-dimensional wax printer (Solidscape, Model Maker II) as described in Manjubala *et al*. (2005). The moulds were then filled with a slurry of HA particles, dried in air and then heated to 600°C to remove the wax moulds. A final sintering treatment was performed at 1300°C for 1 hour (Woesz *et al*. 2005).

### 2.2 Cell culture

Murine pre-osteoblastic cells, MC3T3-E1, were seeded with a density of 80 000 cells cm^−2^ on the surface of the HA plates and cultured in α-MEM (Sigma) supplemented with 10% foetal bovine serum, 30 μg ml^−1^ ascorbic acid and 30 μg ml^−1^ gentamicin in a humidified atmosphere, 5% CO_2_ at 37°C.

### 2.3 Visualization of actin stress fibres

Cell cultures were washed with phosphate buffered saline, fixed with 4% paraformaldehyde, permeabilized with 0.1% Triton-X100 and incubated with 4×10^−6^ M phalloidin–fluorescein isothiocyanate for 30 min at 4°C. Images of the stress fibres were obtained using a confocal laser scanning microscope (Leica).

### 2.4 Quantification of the tissue area

The projected tissue area was determined by transmission light microscopy in combination with image analysis for each channel and time point.

### 2.5 pO2 measurements

Oxygen concentrations were measured using a fibre-optic oxygen micro-sensor with a tip diameter of approximately 30 μm (MicroxTX2, Presens, Regensburg, Germany; Van Dongen *et al*. 2003). The sensor tip was positioned within the channel closely against the growing tissue using a micro-manipulator (M3301, World Precision Instruments, Berlin, Germany) and a stereomicroscope (MZ6, Leica Microsystems, Bensheim, Germany). The measurements were done in a clean bench (Herasafe KS12, Heraeus, Germany) under sterile conditions and the same tissue was used for repeated oxygen measurements at a later time point. These values, expressed as a percentage of air-saturated buffer solution, were plotted against the free area of the central channel.

### 2.6 Modelling curvature-driven tissue growth in two dimensions

Tissue growth is modelled by the motion of the tissue–fluid interface in two dimensions. The initial tissue interface corresponded to the shape of the channel cross-section, with all distances being normalized by the initial channel perimeter. The interfaces were discretized by a set of 90 equally spaced points on the channel surface. The local curvature *κ*_*i*_, at a point *i*, is estimated from the inverse of the radius of curvature of the circle (circumcircle) that passes through this point and its two immediate neighbours, points (*i*−1) and (*i*+1). The tissue at point *i* on the interface is then taken to grow only if *κ*_*i*_≤0 (concave surface). It is assumed that no tissue can be lost, meaning that if *κ*_*i*_>0 (convex surface) at point *i* then the tissue is taken to be quiescent. The tissue at point *i* grows at a rate linearly proportional to the local curvature, i.e. d*s*_*i*_/d*t*=−*λκ*_*i*_, where *λ* is a constant giving the growth rate, with the direction of growth being towards the centre of curvature.

## 3. Results

The impact of three-dimensional geometrical features on new tissue formation *in vitro* was studied in HA plates perforated with channels of different shapes (triangular, square, hexagonal and round) and sizes. HA was chosen as the substrate material, since HA supports the formation of bone tissue rather than other tissue types (Kuboki *et al*. 1998).

### 3.1 Tissue formation *in vitro*

After seeding, the MC3T3-E1 murine osteoblasts adhered to the surface of the HA plates; they started to proliferate, covering the surface of the HA plate and the inner surface of the channels. With ongoing culture time, the multicellular network further amplified and formed new tissue within the channels of the artificial HA plate. The tissue consists of embedded cells and a collagen extracellular matrix. The advantage of the cell line used is that it is able to synthesize collagen fibrils and to build up a collagenous tissue matrix in the presence of ascorbic acid completely autonomously (Rumpler *et al*. 2007). The formation of new tissue started in the corners of the polygonal channels, while cells on the faces were initially resting. Tissue grew uniformly on the surface of the round channels. The tissue thickness in the corners was greater in the triangular channel followed by the square and then the hexagonal channel, that is, in the order of decreasing local curvature. Tissue amplification on the channel faces, however, was greater for the hexagonal channel than the square, followed by the triangular channel. This growth behaviour led to a rounding of the corners and the formation of a round central opening, regardless of the original shape (figure 1*a*). The round opening of tissue is maintained even at the depth of the channels, as verified by confocal laser scanning microscopy (figure 2). With time, the remaining space reduces in diameter, gradually becoming filled by the tissue.

### 3.2 Tissue formation kinetics

Quantitative data on tissue formation kinetics were obtained from two cell culture experiments over more than 37 days with HA plates containing three channel sizes (perimeter *P*=3.14, 4.71 and 6.28 mm) and four different cross-sectional shapes (triangle, square, hexagon and circle). Tissue formation as a function of culture time was estimated by measuring the projected tissue area *A* (figure 3) using transmission light microscopy. The amount of tissue within a given channel is roughly proportional to the projected area *A* at any time point. An effect of pore size on tissue growth has been previously observed and was attributed to the increased surface area available to cells (O'Brien *et al*. 2005). This area dependence can be removed from the data by simply normalizing the projected tissue area *A* by the initial channel perimeter *P*, giving a mean layer thickness of tissue growing on the channel wall. The measured dependence of *A*/*P* on culture time is given in figure 4*a*. There is a significant difference between the mean layer thicknesses due to channel size (perimeter), with more tissue being deposited at any time point in the smaller compared with the larger channels. However, as the mean layer thickness is normalized by the initial perimeter, accounting for the difference in initial surface areas, the effect of channel size implies that other geometric factors control the tissue growth, such as curvature. Although the local tissue formation was different in regions of different curvature (corners versus faces), no significant difference was seen in the total amount of tissue produced as a function of channel shape. This leads to the general effect that the mean layer thickness is similar in the different geometries despite the local variation in tissue distribution within the channels themselves.

### 3.3 Mechanisms of tissue growth

The observations made above illustrate that tissue growth depends on the geometry. One potential mechanism that would give such a behaviour is the generation of hypoxia due to respiratory activity of the cells. It is known, for example, that an exogenous hypoxic state can induce proliferation in mammalian endothelial cells (Schafer *et al*. 2003). A higher growth rate would therefore be expected in the smaller channels. In order to test this hypothesis, the oxygen content was measured in selected channels throughout the whole culture time. No significant correlation was found between the decrease of the central opening and the relative oxygen concentration value during culture time (figure 5) thus excluding the involvement of this mechanistic pathway.

The sub-cellular organization of the tissue-like network in the corners suggests another mechanism behind the geometric control of tissue growth. The orientation of the actin stress fibres within cells neighbouring the tissue border shows a strong parallel alignment with the tissue–fluid interface (figure 6*a*). This is contrasted with the situation on the top of the HA plates outside the channels, where actin stress fibres in the cells show a completely random orientation within the multicellular network (figure 6*b*). It is known that among other factors, internal and/or external forces can trigger the assembly of filamentous stress fibres (Putnam *et al*. 1998; Nelson *et al*. 2005). This might hint towards a mechanism in which mechanical forces arise in cells predominantly in regions of high curvature, i.e. the corners, which in turn stimulates tissue growth.

As a general rule physical systems containing interfaces for which surface tension is important evolve in such a way that the local motion of the interface is controlled by the local curvature (Pimpinelli & Villain 1999). The main driving force behind this is the minimization of interfacial energy via the minimization of interfacial area. A simple two-dimensional mathematical formulation of this assumes that any position on the tissue surface moves normal to the surface by a distance d*s* during a time step d*t*, according to d*s*/d*t*=−*λκ*, where *κ* is the local curvature and *λ* a constant of proportionality. Figure 1*b* shows the numerical solution of this differential equation for the different channel geometries studied. Despite its simplicity, growth is controlled by only one parameter and one proportionality constant, the model gives exactly the same results obtained from cell culture experiments shown in figure 1*a*. In particular, the corners are rounded first (because they have the largest curvature) and the diameter of the central opening reduces subsequently. In the later stages of tissue growth, the holes become circular, meaning the growth rate can be expressed as d*R*/d*t*=−*λ*/*R*, where *R* is the radius of the opening. This implies that the mean tissue thickness *A*/*P* will increase linearly with time as *A*/*P*=2*πλt*/*P*. The growth rate is larger for small perimeters, which is in agreement with the experimental observations (figure 4*a*). The model also predicts that the total tissue area should increase linearly with time at a rate 2*πλ*, which is independent of the starting perimeter. For comparison, *A* is plotted as a function of time for all the experiments (figure 4*b*). However, the experimental data will only superimpose as suggested by the theory if a lag time *t*_0_ is introduced and the tissue area *A* is plotted versus (*t*−*t*_0_). This means that in the cell culture experiments the area increases as *A*=2*πλ*(*t*−*t*_0_) for *t*≥*t*_0_ (figure 4*b* inset). *t*_0_ is an increasing function of the initial perimeter (figure 4*b* inset). This lag time can be understood as the time taken for cells to coat most of the inner channel surface integrating as a coherent network. This happens faster for smaller channel perimeters, corresponding to smaller inner channel surfaces.

## 4. Discussion

Murine osteoblasts are shown to proliferate and form a tissue-like network into the depth of the HA channels, as verified by confocal laser scanning microscopy.

A quantitative analysis of tissue growth within different well-defined channel geometries allowed the evaluation of the effect of geometrical features (channel size and shape) on the tissue formation rate. The total amount of new tissue formed in artificial three-dimensional substrates *in vitro* is surprisingly independent of the shape. It is dependent rather on the channel perimeter with the result that the shorter the perimeter, the more tissue is formed at any time point. Measurements of the oxygen concentration taken over the course of the tissue growth periods illustrate that differences in oxygen deficiency between the channels is not a major factor. As the tissue thickness *A*/*P* is a value that is normalized by the perimeter, this implies that other geometrical factors control tissue growth.

The simple model for curvature-driven growth can describe the experimentally observed growth behaviour remarkably well, considering that it uses just one known, geometric variable (the surface curvature) and one unknown parameter of the growth rate (figure 1). Tissue growth always started in the corners of the channels (large local negative curvature), while cells on the channel faces (zero curvature) produced tissue only when their neighbourhood became curved from tissue growing outward from the corners. The curvature-driven tissue growth is also consistent with the experimentally measured independence of the average tissue thickness to the channel shape at constant perimeter length. If the assumption that the local growth rate is proportional to local curvature is correct, then the rate of change of average tissue thickness will be proportional to the average curvature. Following Fenchel's theorem (Fenchel 1929), the average curvature, *K*, of any closed convex plane curve is 2*π*/*P*, where *P* is the perimeter. All the shapes investigated can be described in two dimensions by closed convex plane curves, meaning that the average tissue thickness should be the same at fixed perimeter, as observed experimentally. In addition, this also implies that smaller channels, which have higher average curvatures, will have a higher tissue thickness, as is observed. The preceding discussion clearly shows that curvature-driven growth corresponds well with the experimental results. The physics behind curvature-driven growth is well understood for membrane mechanics, crystal growth and phase transformations. The driving force in these physical systems is the surface tension that tends to reduce the surface (and therefore its curvature) as much as possible for a given volume. It is not obvious how to link this to the behaviour of cells and their cytoskeletons, but the observation of stress fibres provides a hint. The strong alignment of the stress fibres with the tissue interface, as well as their high concentration in the corners, suggests that mechanical forces may develop within the tissue. If there is a tensile cell–cell interaction between neighbouring cells in the tissue growth front, this could simulate a physical surface tension as in the physicochemical systems mentioned above. Indirect evidence supporting the idea that mechanical forces are responsible for the curvature-driven growth comes from the work of Nelson *et al*. (2005), in which it was shown on flat substrates that the cell proliferation is higher in regions of high force. Of course, this idea is still quite speculative and requires further work to be ascertained.

The curvature-driven growth is a process which leads to self-organization, in that tissue amplification creates a local change in curvature which in turn feeds back to the cells themselves. As a consequence, the curvature-driven growth will cause channels or holes to be filled, which has implications in many areas of bone biology and medicine. This effect may be the driving force for the filling mechanism of resorption lacunae by osteoblasts after osteoclastic bone resorption by reducing the curvature of the lacunae to zero (Robling *et al*. 2006; Seeman & Delmas 2006). The size dependence of the lag time in the tissue growth process, as well as the size dependence of curvature, points towards a critical size above which closure of the gap would take too long to occur on a physiological time scale. For example, the channels with a diameter of 2 mm showed a strongly reduced tissue growth within five weeks. This is reminiscent of the existence of a critical defect size in bone above which defect healing no longer occurs (Goldstein 2000; Fratzl & Weinkamer 2007).

Another aspect of our results is that single cells with a size of the order of a few tens of micrometres can ‘feel’ the radius of curvature on the scale of millimetres. Where cells were stained for actin, it becomes apparent that cells interact through their cytoskeleton. Actin stress fibres between neighbouring cells in the tissue border are aligned parallel to the tissue–fluid interface in such a way as they appear as a single ring-like structure (figure 6*a*). However, on the top of the HA plates outside the channels, actin stress fibres are randomly oriented (figure 6*b*), suggesting the absence of directed mechanical forces. Even before the ring-like structure is formed, actin stress fibres aligned parallel to the inner tissue surface can be seen in the channel corners. The observation of strongly oriented stress fibres parallel to the tissue–fluid interface gives an indication of the potential mechanism behind the tissue growth. Such stress fibres may be associated with the interaction of neighbouring cells via mechanical forces (Chen *et al*. 2004; Kaunas *et al*. 2005). In addition, the simulations of curvature-driven tissue growth are based on the assumption that the system evolves to minimize surface tension, which is the highest in the regions of high curvature. Using this assumption, the experimental growth kinetics of the tissue interface can be simulated, which is indirect evidence that lateral stress in the near surface region is responsible for growth kinetics. A similar behaviour was also observed by Nelson *et al*. (2005), which supports this interpretation. They observe that cell proliferation correlates with regions of high mechanical forces. Finite-element simulations also show that the high force concentrations occurred in regions of high curvature.

A growing body of evidence suggests that mechanical events, in addition to molecular biological and chemical ones, play a central role in the process where cells explore and react to their neighbourhood. Even in two-dimensional multicellular systems, local mechanical forces appear in defined regions where cell proliferation is also increased compared with the neighbourhood (Stegemann & Nerem 2003; Nelson *et al*. 2005). Following this mechanism, cells lying on the faces of the channels start proliferating only when they get a mechanical stimulus from being integrated into the tissue network growing out from the corners. In addition, the dihedral corners may not only be spatially optimized for force generation, as it is known that cells more closely mimic the *in vivo* environment when they are able to develop focal adhesion sites in three dimensions as opposed to flat substrates (Cukierman *et al*. 2001, 2002; Geiger 2001; Beningo *et al*. 2004). Mechanical equilibrium requires that stress fibres are either attached to the wall (corners) or form closed loops (as observed in the ring-like structures). These rings are also reminiscent of the ‘purse strings’ reported in embryonic tissue healing, in the form of actin bundles aligned with the wound margin (Bement *et al*. 1993). Thus, the wound is closed partially by active mechanical contraction (Martin & Lewis 1992; Jacinto *et al*. 2001). It seems that connective tissues may use a highly conserved set of signals and cytoskeleton-based mechanisms to close holes in artificial systems.

## 5. Conclusion

In this paper, quantitative measurements were made of the growth kinetics of collagenous tissue in an osteoblast culture for different three-dimensional channel geometries. Parameters describing channel geometry, namely the channel surface area (related to perimeter) and local curvature, have been shown to strongly influence the tissue growth rate. Simple two-dimensional simulations support the idea of curvature-driven tissue growth, that is, the amount of tissue deposited is proportional to the local curvature. The awareness that a basic geometrical parameter, namely channel curvature, affects tissue formation kinetics in a three-dimensional environment has far-reaching implications in the understanding of bone remodelling and defect healing as well as for scaffold design in bone tissue engineering.

## Figures and Tables

**Figure 1:**
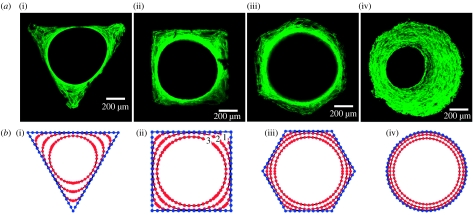
(*a*) New tissue formed in three-dimensional matrix channels. Actin stress fibres are stained with phalloidin-FITC and visualized under a confocal laser scanning microscope. Here, the tissue formation is shown (i–iii) after 21 days and (iv) after 30 days of cell culture in the channels of a (i) triangular, (ii) square, (iii) hexagonal and (iv) round shape introduced into a HA plate *in vitro*. (*b*) Numerical simulation of tissue formation within channels of various shapes: (i) triangular, (ii) square, (iii) hexagonal and (iv) round. The lines (early time point 1, ongoing times 2 and 3) mark the simulated development of tissue formation due to ongoing culture time, which corresponds closely to the observed development of new tissue formation *in vitro*.

**Figure 2:**
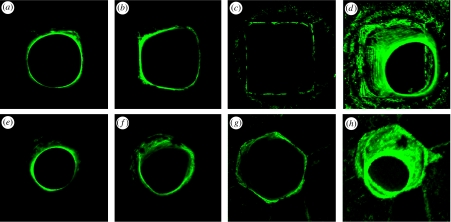
Three-dimensional fluorescence images. The cytoskeleton of the cells within the tissue is visualized by FITC staining and serial two-dimensional sections were obtained with a confocal laser scanning microscope. Three-dimensional pictures were then stacked from all single pictures. In (*a*–*c*) three examples of the serial pictures obtained from the tissue formed in a square channel are shown at depth (*c*) 0, (*b*) −135 μm and (*a*) −470 μm, as well as (*d*) the combination of all single pictures. In (*e*–*g*) three examples of the serial pictures obtained from the tissue formed in a hexagonal channel are shown at depth (*e*) −485 μm, (*f*) −260 μm and (*g*) 0, as well as (*h*) the combination of all single pictures.

**Figure 3:**
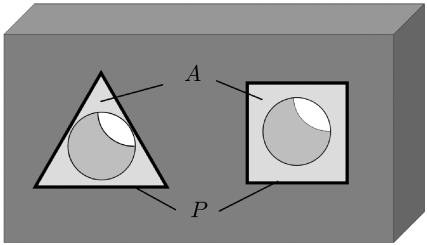
Sketch of two channels in a HA plate (dark grey). The figure illustrates the definition of the initial perimeter of the channel cross-section *P* and of the projected tissue area *A* (light grey) with transmission light microscopy in each channel, which was used to calculate quantitative data for tissue formation kinetics.

**Figure 4:**
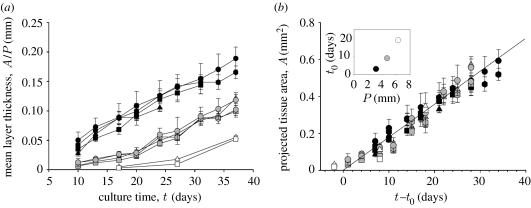
Quantitative tissue formation and kinetics. (*a*) Mean tissue layer thickness (calculated by the normalization of the projected tissue area to the perimeter) as a function of culture time in different shapes (illustrated by the appropriate symbols) and different channel perimeter *P*=3.14 mm (black), 4.71 mm (grey) and 6.28 mm (white). Data represent the mean value+s.e.; *n*=10. (*b*) Projected tissue area as a function of culture time minus lag time, which is the time that is needed until cells start curvature-driven growth. In the inset, the lag time is given as a function of initial channel perimeter.

**Figure 5:**
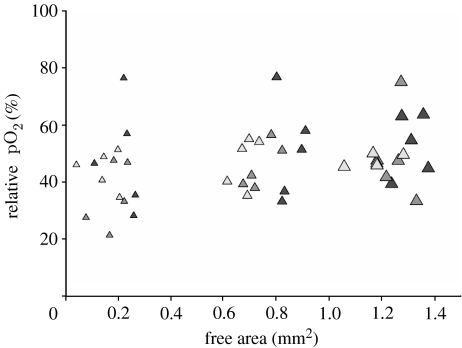
pO2 measurements. MC3T3-E1 cells were seeded onto the HA and tissue formation was running over a time period of 43 days. At distinct time points, 25 days (black), 32 days (dark grey) and 43 days (light grey), the oxygen concentration inside the central channel was measured in the direct vicinity of the growing tissue. These values, expressed as percentage of air-saturated buffer, were plotted against the free area of the central channel. The size of the triangle indicates the hole size (small, *P*=3.14 mm; medium, *P*=4.71 mm; and large, *P*=6.28 mm); *n*=5.

**Figure 6:**
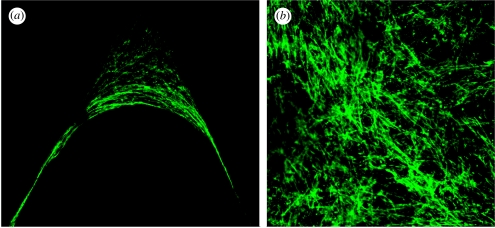
Fluorescence microscopic images of the stained cytoskeletal stress fibres within the new formed tissue. (*a*) A zoom into the corner of a triangular HA channel shows a strong orientation of the stress fibres parallel to the tissue surface. (*b*) An image of the cell network on the external flat surface at regions between the channels shows a completely random orientation.
